# Interspecific Communicative and Coordinated Hunting between Groupers and Giant Moray Eels in the Red Sea

**DOI:** 10.1371/journal.pbio.0040431

**Published:** 2006-12-05

**Authors:** Redouan Bshary, Andrea Hohner, Karim Ait-el-Djoudi, Hans Fricke

**Affiliations:** 1 Department of Zoology, University of Neuchâtel, Neuchâtel, Switzerland; 2 University of Cambridge, Department of Zoology, Cambridge, United Kingdom; 3 Max-Planck-Institut für Verhaltensphysiologie Seewiesen, Starnberg, Germany; 4 Leibniz-Institut für Meereswissenschaften (GEOMAR), Universität Kiel, Kiel, Germany; Emory University, United States of America

## Abstract

Intraspecific group hunting has received considerable attention because of the close links between cooperative behaviour and its cognitive demands. Accordingly, comparisons between species have focused on behaviours that can potentially distinguish between the different levels of cognitive complexity involved, such as “intentional” communication between partners in order to initiate a joint hunt, the adoption of different roles during a joint hunt (whether consistently or alternately), and the level of food sharing following a successful hunt. Here we report field observations from the Red Sea on the highly coordinated and communicative interspecific hunting between the grouper, *Plectropomus pessuliferus,* and the giant moray eel, Gymnothorax javanicus. We provide evidence of the following: (1) associations are nonrandom, (2) groupers signal to moray eels in order to initiate joint searching and recruit moray eels to prey hiding places, (3) signalling is dependent on grouper hunger level, and (4) both partners benefit from the association. The benefits of joint hunting appear to be due to complementary hunting skills, reflecting the evolved strategies of each species, rather than individual role specialisation during joint hunts. In addition, the partner species that catches a prey item swallows it whole immediately, making aggressive monopolisation of a carcass impossible. We propose that the potential for monopolisation of carcasses by one partner species represents the main constraint on the evolution of interspecific cooperative hunting for most potentially suitable predator combinations.

## Abstract 2

Videos and field data reveal that the grouper and the giant moray eel cooperate to hunt together, each taking on different roles. Such cooperation has only been observed in mammals and birds.

## Introduction

Cooperative hunting, i.e., the increase in successful prey capture observed when two or more individuals engage in a hunt, has been demonstrated in a wide variety of species [1–4]. In many cooperatively hunting species, hunts can best be described as opportunistic, simultaneous individual hunts [4], in which each animal tries to maximise the probability of catching the prey for itself. True coordination, as defined in [5], exists only if individuals play different roles during a hunt. Role differentiation implies that individuals will adopt roles that have a lower probability of personal success or a higher risk of injury than other roles would offer, e.g., hunts where some individuals act as chasers while others block the escape routes of prey. Such coordination is known for only a handful of species [5–8], all of which are mammals or birds. Individual role specialisation within coordinated hunts is even more rare and has only been observed in two studies to date [7,8]. Communication between group members to initiate a coordinated search for suitable prey (for which the term “intentional hunting” has been used) is known only from a single population of chimpanzees [5]. The same population of chimpanzees is also well known for respecting prey ownership, where the successful individual shares with cohunters [5]. While simultaneous feeding on a prey carcass may also occur in carnivores, access in these species is best predicted by individual rank and/or nepotistic toleration of related lower ranking individuals [4].

Here we describe interspecific and communicative hunting between the grouper, *Plectropomus pessuliferus,* and the giant moray eel, *Gymnothorax javanicus,* observed in the coral reefs of the Red Sea. Groupers are diurnal predators, whereas the morays are nocturnal hunters and usually rest in crevices during the day. The hunting strategies of the two predators are also very different. Groupers are semi-benthic piscivors, which hunt in open water. In order to avoid predatory groupers, reef fish hide in corals (apart from pelagic prey like fusiliers). Moray eels, in contrast, sneak through crevices in the reef and attempt to corner their prey in holes. Consequently, the best strategy for prey to adopt in order to avoid moray predation is to swim into open water. The hunting strategies of the two predators are therefore complementary, and a coordinated hunt between individuals of the two species confronts prey with a multipredator attack that is difficult to avoid [9]; prey are not safe in open water because of the grouper hunting strategy but cannot hide in crevices because of the moray's mode of attack.

Here we first provide some descriptive information on the interactions between the two predators (i.e., frequency, duration, and distance between partners during a joint hunt) and use a simplified version of Waser's gas model [10] to show that associations are not due to random encounters. Second, we describe the signals produced by the groupers that serve to elicit joint hunting. Third, we present experimental evidence that the production of these signals is inhibited if the grouper is satiated. Finally, we present observational evidence that both partners increase their hunting success when they are in association. We then discuss the selective conditions that might promote such an unusual interspecific cooperation.

## Results

### Evidence That Associations Are Nonrandom

**Figure 1:**
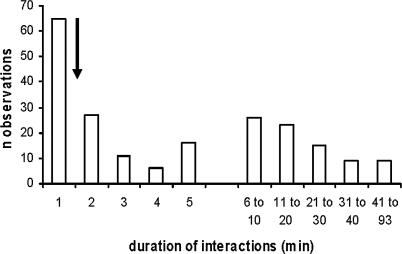
Many Associations between Groupers and Moray Eels Are Longer than Expected by Chance Observed frequency distributions of durations (min) of interactions between groupers and moray eels. The *x*-axis shows different time categories that were grouped in a non-linear fashion. The arrow almost above the 2 min time category indicates the average duration of associations (100 s) predicted by a null model, assuming independent movements of individuals of the two species.

Because the exact number of moray eels in our study area is unknown, we compared the distribution of observed durations of associations with the value predicted from a simplified version of Waser's gas model as an acceptable compromise [10]. The gas model yields mean association durations based on the assumption of independent movements (see Methods). Associations ranged from less than 1 min up to a value of 93 min. A proportion of observed durations of interactions fit the null hypothesis of random association, which predicts durations of 100 s. However, 56% of the 207 interactions lasted longer than predicted by the null hypothesis, among which 71% lasted at least three times longer (Figure 1).

**Figure 2:**
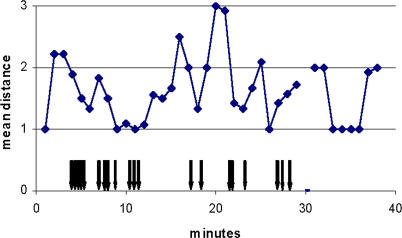
The Two Predators Remain near Each Other During Joint Hunts Mean distance given as multiples of grouper body length (estimated 70 cm) between a grouper and a moray eel per minute association, analysed on screen from a 38-min film clip of a joint hunt that was already ongoing when the camera man joined. The gap in the data is due to the camera man focussing the lens on one individual such that nothing else would be seen on screen. Arrows indicate the timing of grouper signalling.

Figure 2 shows the average distance between a grouper and a moray eel per minute of joint hunting for a single observation, where video filming allowed detailed analysis of this variable. The two partners stayed together at a distance of between 1 and 3 times 70 cm (which is one approximate grouper body lengths) over a period of 38 min.

### Signals Produced by the Groupers

Groupers actively visit moray eels at their resting places and make use of visual signals to engage morays in a joint hunt. This involves shaking the head at high frequency (3–6 shakes per second) directly in front of the moray eel, usually few centimetres away from the moray's head (Video S1). During head shakes, the soft part of the dorsal fin is erect while the bony part is flat, apart from short-term flickers of approximately 0.1–0.3 s duration. All 14 of the groupers in our study produced this head-shaking signal at least once during observations. In 58% (*n* = 120) of observations, the morays responded to head shaking by leaving their crevices, and the two fish then swam off through the reef (Video S2). Moray eels were never observed to signal to groupers.

The joint activity of the two fish, measured from the moment the moray left the crevice until the moray re-entered a crevice and did not re-emerge, ranged from a few seconds up to 44 min. Joint movement was often interrupted, because moray eels could remain in a crevice for several minutes before moving on. Groupers often repeated the head-shaking signal in these situations (Figure 2, arrows). Groupers did not always signal to morays when they visited them; groupers could simply pass nearby and/or lay down on the sand next to a moray. However, joint hunting was significantly more likely to occur if a grouper signalled (70 out of 120 observations) than if it did not signal (11 out of 38 observations) (χ^2^ = 8.4, *n*=158, degrees of freedom = 1, *p* < 0.01).

In addition to signalling before any hunting events, groupers also recruited moray eels after unsuccessful hunts. Typically, a hunt ended unsuccessfully because prey escaped into a crevice that was inaccessible to the grouper. In the majority of observations, the groupers either swam off immediately without further hunting attempts, or they remained nearby, usually above the hole but out of sight. This latter behaviour usually lasted several minutes and sometimes led to a second hunt, presumably involving the same prey fish. In a few cases, however, the groupers (*n* = 9 individuals, total observation = 14) swam to a giant moray eel that was within 15 m of the prey's hiding place, signalled to the moray, and apparently tried to guide it to the prey's location. Finally, six groupers (total *n* = 21) were observed to remain directly above the crevice where prey were hiding and engage in wide head shakes, while simultaneously performing a head stand. The dorsal fin was used as in the standard head-shake signal to the moray, but the head movements differed, most obviously because of the pauses between single shakes (Video S3). Headstand shakes invariably attracted other predators to the prey's location in the crevice. On ten occasions, a moray eel joined the grouper and explored the crevices (Video S4). Napoleon wrasses, *Cheilinus undulatus,* approached and inspected the hiding place on ten occasions, and a yellowlip emperor, *Lethrinus xanthochilus,* approached on one occasion. Guiding moray eels to hidden prey and signalling above hidden prey to attract other predators resembles the behaviour of honey guides that catch the attention of badgers or humans and guide them to bee nests [11]. The badgers or humans raid the nest and the bird is then able to feed on what remains.

### Signalling Is Motivated by Hunger

Six individual groupers received a fish at the onset of an observation session, and we therefore knew that their hunger level was likely to be lower than during an average random observation. The groupers did not signal to a moray eel during the 120-min observations following the consumption of the prey. Because all six individuals were observed to signal to morays during other observation sessions, they were significantly less likely to signal to moray eels after having eaten than during protocols where their hunger levels were unknown to the observer (Wilcoxon test, *n* = 6, *T* = 0, *p* = 0.032).

### Joint Hunting Appears to Be Mutualistic

In association, groupers were significantly more successful at hunting than expected, based on time spent in association (Binomial test, *n* = 16, *p* = 0.007) (Table 1). The analysis does not control for individual contribution to the dataset. Five different individuals were successful in presence of moray eels and eight individuals were successful in the absence of moray eels (three individuals appear in both datasets). In association, groupers caught almost five times as many prey items per unit time than when morays were absent (0.19 preys h^−1^ with moray to 0.04 preys h^−1^ without moray). The ten occasions on which groupers hunted successfully without a moray included one observation of two groupers hunting the same prey simultaneously and two observations of a grouper hunting with a napoleon wrasse. The wrasse seems to fulfil a similar function to the moray eel; Although it cannot enter crevices where preys are hiding, it can often destroy hides with its powerful jaws.

**Table 1:**
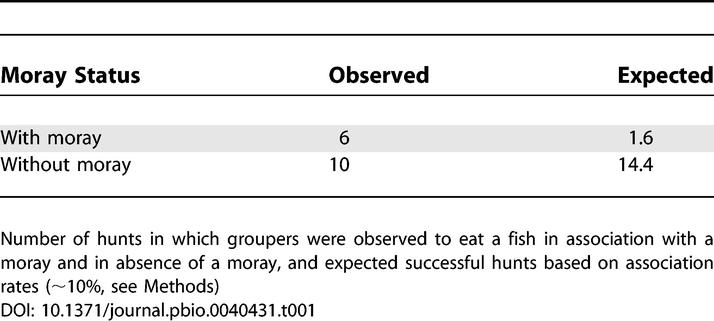
Groupers Benefit from Joint Hunting

On five occasions, we observed moray eels catch prey when hunting with a grouper. Thus, the hunting success of both predators was roughly equivalent. Moray eels were never observed to hunt successfully when solitary. This is not surprising, however, as moray eels observed alone simply remained in their crevice and did not appear to search for prey. Therefore, statistical analysis seems inappropriate because solitary morays were merely resting. Nevertheless, the success rate of 0.36 prey h^−1^ (5 prey in 829 min of coordinated movement with groupers) is quite efficient in comparison to groupers (0.19 prey h^−1^, see Methods).

During the 31.8 h of association between groupers and morays, we never observed any overt aggression from either partner towards the other. This includes the 11 observations of successful hunts. The individual fish that caught the prey swallowed it quickly and whole without any aggression from the unsuccessful partner.

## Discussion

We have presented several lines of evidence for interspecific communicative, coordinated, and cooperative hunting between two species of reef fish predators. We found the following: (1) individual groupers and moray eels frequently spent more time in association than predicted by a null model of chance encounters, (2) groupers actively signalled to elicit joint hunting and to recruit moray eels, (3) satiated groupers did not signal, and (4) both partner species increased their hunting success in association. Although the evidence for groupers is straightforward, the typical nightly activity pattern of moray eels precluded any detailed statistical comparison of hunting success data. However, given that moray eels showed a higher success rate than groupers in association (0.36 prey h^−1^ versus 0.19 prey h^−1^), and only caught fish with groupers during daytime, we can at least conclude that moray eels broaden their hunting activity to daylight hours due to their interactions with groupers. We did not detect any behaviour of either partner that might have served to increase the hunting success of the other species at the expense of its own hunting success. In other words, altruistic behaviour did not seem to occur during joint hunting events. Thus, the outcome of joint hunting between the groupers and the moray eels appears to be a by-product mutualism [12].

Intraspecific coordinated hunting with role differentiation between individuals is known for only a handful of species [5–8] and has not yet been observed in fish, although simultaneous group hunting exists [13]. The evolution of coordinated hunting may occur only rarely because several problems related to the coordination of behaviour must be overcome. For example, individuals must perform actions during coordinated hunts that are successful only if they are accompanied by complementary actions performed by other individuals at the same time. Consequently, cognitive constraints may limit species' ability to coordinate in this manner. In addition, the adoption of different roles during coordinated hunts means that certain individuals will experience a reduced probability of catching prey themselves when hunting and so incur a net cost, whereas other roles are associated with increased rates of prey capture, and therefore yield a net benefit for the individuals' playing these roles. Hence, intraspecific coordinated hunting is linked to the well-known evolutionary problems of unequal payoffs and potential defection that are associated with altruistic behaviour. Tit-for-tat–like role alternation [14] or the sharing of prey [5] are potential solutions to this problem. Finally, phylogenetic constraints may limit the kind of roles that individuals are able to adopt, and so reduce the likelihood of successful coordinated hunting. For example, a lioness will never be able to run with the speed of a cheetah, even though this ability would help in a coordinated hunt.

Interspecific cooperation, as we have observed between groupers and morays, seems to overcome the difficulties of intraspecific coordinated hunting. Any potential cognitive constraints regarding the division of roles are absent, because associating partners behave in exactly the same manner as they do when hunting alone. Each player uses only its evolved hunting strategy, and there is no pressure to learn specific new behaviours that yield advantages when they form part of a coordinated effort. In addition, both grouper and moray apparently try to maximise their individual capture rates, with mutual benefits accruing simply from the joint movements that serve to amplify their individual predation efforts. Interspecific coordinated hunting of this kind can emerge, therefore, from a simple associative learning process, whereby each species associates increased rewards with hunting in the vicinity of the other species. The same mechanism may explain the many commensal associations in coral reef fish, where individuals of one species follow a so-called nuclear species [15–17]. However, these associations lack the signalling and mutually coordinated movements observed in grouper–moray interactions.

At present, we can only speculate about the nature of the information that is conveyed by grouper head-shake signals. We have never observed head shaking of this kind, or anything similar, in moray eels, so it seems unlikely that grouper signals represent the generalisation of the morays' natural intraspecific repertoire to an interspecific context. The simplest explanation, therefore, is that these signals indicate only the motivation of the grouper to engage in hunting, which then becomes positively associated with hunting success for the moray eels. That is, head shakes may act as a form of conditioned stimulus for the morays. This is particularly likely in situations where a grouper has already cornered a vulnerable prey fish, allowing morays easily to associate head shaking by the grouper with their own hunting success, or at least a close encounter with a prey.

Active signalling between partners to initiate joint hunting (rather than merely indicating the presence of a food source as in the honeyguide example [11]) has been interpreted as a major cognitive achievement in chimpanzees [5]. Only a single West African population, in Taï National Park, Ivory Coast, is known engage in such behaviour; all other chimpanzee populations hunt more opportunistically, following chance encounters with monkey prey [5,18,19]. Such differences have generated great interest in the anthropological literature because of the disputed importance of complex cooperative hunting for early human evolution [20–22]. Whether these differences between populations reflect true cognitive differences rather than mere differences in ecology remains to be tested. Our results suggest the latter, unless future studies are able to demonstrate more convincingly that complex cognitive processes underlie chimpanzee communication during hunt initiation. In our study system, signalling by groupers seems to be a necessary adjustment to the morays' activity pattern. Because moray eels are usually inactive during daytime, groupers cannot join a partner that is already looking for prey but must induce a moray to become active.

We hypothesise that swallowing prey whole, as we observed in the groupers and moray eels, is an important condition for the occurrence of interspecific cooperative hunting. In principle, interspecific hunting could also improve the hunting success of mammalian predators, but it is rarely found, and examples always involve humans as one of the partners [23,24]. Pursuit hunters, like hyenas, that coordinate their hunts with a speed predator, like a cheetah, or a sit-and-wait predator, like a leopard, could also increase their hunting success in the same way as groupers and moray eels. However, after successful hunts, competition would arise immediately over which species feeds first and which parts of the carcass are eaten, and problems of cheating and defection would be rife. Consequently, we propose that the defensibility of a kill is the decisive obstacle preventing the evolution of interspecific cooperative hunting in mammals and other taxa. If cheating after a successful hunt is not an option, however, interspecific cooperative hunting may readily evolve. Moreover, we suggest that the multiple predation effect of interspecific cooperation is the key to overcoming the inherent problem presented by the joint hunting of nonsharable prey; namely, overall prey capture rate needs to be at least twice that obtained by a solitary hunter in order to yield net benefits [4]. In accordance with our hypothesis, we have two observations, one video-documented (unpublished data), of lunartail groupers, *Variola louti,* which signalled once to a grey moray eel and once to a giant moray eel in a very similar way to our study groupers [25]. The latter happened after we had first attracted the grouper with a dead fish and then hidden the fish in a crevice near the moray. We expect to find other species combinations that hunt cooperatively, but only in situations where there is no competition over carcasses.

## Materials and Methods

### Study site and subjects.

All observations were made between September 2002 and December 2004 in the eastern part of Mersa Bareika, Ras Mohammed National Park, Egypt. Along the 2800-m coastline, 14 groupers, varying between 55 and 100 cm in total length (estimation) were recognised by individual spot patterns on their body. Individuals were followed by snorkelling observers for up to 180 min. All relevant information was noted with a pencil on Plexiglas plates. In a subdivision of the total study area (700 m of coastline), seven moray eels, varying between 130 and 200 cm in total length (estimation), were also individually known by their spot patterns. However, individual identity often could not be verified while following the groupers.

### Association patterns.

Groupers were observed for a total of 406.4 h. We used this total observation time to calculate the proportion of time spent in association with moray eels. We defined an association as a grouper and a moray being less than 10 m apart from each other. This is a very conservative distance criterion for our calculations (in the sense that it favours the null hypotheses), because the partners are usually much closer to each other during a joint hunt (see Results and Videos S1–S4). Moray eels were observed in the same manner for 18.5 h. Because moray eels are crepuscular and nocturnal hunters, individuals did not move during these observations. We therefore concentrated on the groupers. However, we have an additional >50 h of observations focussing on moray eels with either HF or Jürgen Schauer (Leibniz Institut für Meereswissenschaften [GEOMAR], Kiel, Germany) sitting in front of moray eels for filming purposes. With one exception, morays never moved during these film sessions and were not visited by groupers. We do not know whether these results reflect natural encounter rates with groupers from a moray perspective or whether the data are biased due to the presence of humans.

### Application of Waser's gas model to the grouper–moray associations.

In brief, the model uses the average velocities of two groups or individuals in a 2-D space, plus a criterion for maximal distance, to calculate mean durations of associations [10]. Because we noted the movements of groupers only relative to the coastline, our calculation simplifies to a 1-D space, which yields lower swimming speeds and hence increases association durations predicted by the null hypothesis. The calculations further simplify because moray eels rarely, if ever, moved and can be assumed to be stationary objects. The crucial determinant of association durations predicted by the null hypothesis is therefore the swimming speed of groupers relative to the coastline and our association criterion of 10 m. We constructed a map of the coastline and measured distances between key landscape points. We noted the position of a grouper relative to the coastline every 15 min and, in addition, we noted each time a grouper changed direction. This information combined with the duration of each protocol allowed us to calculate the average swimming speed of each grouper relative to the coast line. The average speed used for the null model is the average speed of all groupers (one value per grouper, *n* = 14 individuals, unpublished data). The predicted mean association duration of the simplified model is the time it takes a grouper on average to swim 20 m, i.e., 100 s.

### Signalling by groupers and average distance between partners during joint hunts.

To test the effects of feeding on grouper signalling, we allowed six individuals to eat a fish (purchased at the local market) at the onset of an observation session and then followed them for 120 min and recorded their behaviour following our standard observational protocol. We then compared these data with our recordings of the same individuals during observation sessions where we did not know their hunger state (matched pair design). To analyse the signals in more detail and to document the behaviours of groupers and moray eels, we filmed interactions between groupers and moray eels (all successful sequences were made following a grouper, see above). Illustrations are provided in Videos S1 and S3. In addition, we observed one 38-min sequence of a joint hunt, from which we were able to analyse on-screen the distance between the two partners during the hunt, measured as multiples of grouper body length.

To calculate hunting success of groupers in association and alone, we only considered protocols where groupers were observed to hunt at least once. In addition, once a grouper had caught a fish during an observation session, the rest of the protocol was discarded, because hunting success stopped any further hunting efforts. A total of 286 h of observations were available. The groupers spent approximately 11% of that time in association with moray eels (31.5 h out of 286 h). The small sample size of observed successful hunts (*n* = 16 successful hunts performed by 10 different individuals) precluded statistical analysis at the individual level. This is because any analysis where the null hypothesis deviates from a 50:50 distribution (in our case it is roughly 10:90) is only useful if the number of observations is sufficiently high that predicted values for the rare situation is at least one unit. In our case, an individual with one observed successful hunt would be most likely to have achieved that success when on its own, and the most likely theoretical outcome for hunting success in association is zero. We therefore analysed all data together using a binomial test with a truncated expectation (10% of all successful hunts observed in association).

The hunting success of moray eels could not be analysed in the same way as the grouper data, because solitary morays were obviously not hunting. To calculate their hunting success rate in association, we took into account the fact that morays often did not react to grouper visits by increasing their activity or they stopped searching for prey while the groupers were still motivated. Thus, groupers spend a large proportion of their time in association signalling to or waiting next to moray eels that did not leave their crevice. This time must be included to calculate grouper hunting efficiency in association, but not in the calculations for moray hunting efficiency. For the morays, we summed the number of times from first moray movement to the last moray movement during each joint hunting event. The total number of prey caught by morays during association was divided by this value to calculate the morays' hunting success rate.

## Supporting Information

Video S1 A Grouper Signalling to a Moray Eel Resting in a Cave (1.1 MB WMV)

Video S2 Grouper and Moray Eel Swimming off Together after the Grouper Signalled (2.0 MB WMV)

Video S3 A Grouper Performing a Headstand Shaking of Its Head above the Hiding Place of a Prey That Escaped the Hunt (2.1 MB WMV)

Video S4 A Moray Approaches the Place Where the Grouper Performs Its Headstand Shaking (2.1 MB WMV)
